# Targeted next-generation sequencing for pediatric lower respiratory tract infections: a retrospective study

**DOI:** 10.3389/fcimb.2025.1652949

**Published:** 2025-08-28

**Authors:** Linlin Li, Jing Guo, Jielin Chen, Jiangyang Zhao, Jiahui Liang, Minxue Liu

**Affiliations:** ^1^ Medical Science Laboratory, Children’s Hospital, Maternal and Child Health Hospital of Guangxi Zhuang Autonomous Region, Nanning, China; ^2^ Department of Pediatric Respiratory Medicine, Children’s Hospital, Maternal and Child Health Hospital of Guangxi Zhuang Autonomous Region, Nanning, China

**Keywords:** pediatric, lower respiratory tract infections, bacterial culture, qPCR, tNGS

## Abstract

**Objectives:**

Lower respiratory tract infections (LRTIs), including bronchitis and pneumonia, are common pediatric conditions. Accurate and timely pathogen identification is essential in this population, with targeted next-generation sequencing (tNGS) significantly improving detection rates. This study aimed to systematically assess the clinical utility of tNGS in identifying pathogens in pediatric LRTIs.

**Methods:**

A retrospective analysis of 107 pediatric patients with lower respiratory tract infections (LRTIs) was conducted between January 2024 and December 2024. The concordance of tNGS, quantitative polymerase chain reaction (qPCR), and microbial culture results with clinical diagnoses was assessed. Data were analyzed using the Statistical Package for the Social Sciences (SPSS), with statistical significance set at *P* < 0.05.

**Results:**

Of the 107 pediatric patients, 34 (31.8%) had single-pathogen infections while 73 (68.2%) had multiple infections, with bacterial-viral co-infections comprising 42.5% of the latter group. The tNGS results demonstrated a concordance rate of over 66% with clinical diagnoses, which was significantly higher (P < 0.001) than that of qPCR and culture. The analysis revealed 80% concordance between tNGS and qPCR results. Notably, tNGS demonstrated 90% concordance with culture methods in approximately 70% of comparative detections. Furthermore, for *Human rhinovirus* and *Mycoplasma pneumoniae*, the RPM values for tNGS were significantly higher (P<0.05) in (tNGS+qPCR+) samples than in (tNGS+qPCR−) samples. Compared with *Human rhinovirus* (AUC=0.759), *Mycoplasma pneumonia*e exhibited stronger discriminatory power (AUC=0.917).

**Conclusion:**

The tNGS results demonstrated high concordance with the clinical diagnosis, supporting its high applicability in diagnosing pathogens in pediatric patients with severe, mixed, or refractory infections.

## Introduction

Lower respiratory tract infections (LRTIs) represent a major global health challenge, significantly contributing to morbidity and mortality worldwide, particularly among children (GBD 2019 LRI Collaborators, 2022). In 2016, LRTIs were associated with 652,572 deaths among children under 5 (GBD 2016 Lower Respiratory Infections Collaborators, 2018). LRTIs were caused by diverse pathogens, including bacteria, viruses, mycoplasmas, fungi, and parasites, with respiratory viruses accounting for a significant proportion of cases (Liu et al., 2018; Wang et al., 2020). Major causative agents include influenza, respiratory syncytial virus (RSV), human metapneumovirus (hMPV), enterovirus, rhinovirus, parainfluenza virus (PIV) 1–4, adenovirus, and more recently SARS-CoV-2 being the most prevalent (Burrel et al., 2021; Chu et al., 2022). Since 2021, shifts have occurred in the epidemiology and seasonal patterns of these pathogens (Contes and Liu, 2025). For instance, RSV peaked 1–2 months earlier, and the Southern Hemisphere experienced a longer interval between peaks, with the first wave peaking earlier than usual (Memish et al., 2020). Clinically, viral LRTIs often present with more gradual symptom onset compared to bacterial infections, so in the absence of accurate pathogen identification, clinicians are frequently compelled to resort to empirical antimicrobial therapy. This practice not only increases the risk of inappropriate antibiotic use but may also contribute to the emergence and spread of resistant bacterial strains, adversely affecting clinical outcomes. Pathogen-specific infection control and treatment are required, highlighting the importance of identification in pediatric LRTIs.

Bronchoalveolar lavage fluid (BALF) and sputum are clinically relevant samples for detecting pathogenic microorganisms in pediatric LRTIs (Wei et al., 2023). Conventional detection methods typically include microbial culture, smear microscopy, and qPCR. Microbial culture remains the gold standard for microbiological identification. However, this method suffers from being time-consuming, insensitive, and ineffective, especially when applied to viruses or difficult-to-culture organisms (Millot et al., 2017). While qPCR is fast and precise, its application is limited by the need for pathogen-specific primer/probe design and low multiplexing capability in a single assay (Gauthier et al., 2023).

Metagenomic next-generation sequencing (mNGS) technology is increasingly employed for detecting, identifying, and analyzing human pathogens (Gu et al., 2019). By enabling simultaneous sequencing of billions of DNA fragments, mNGS provides an unbiased method to identify both known and novel pathogens, potentially even discovering previously unknown organisms (Shi et al., 2022). In recent years, mNGS has provided an even more efficient and accurate means of pathogen detection, revolutionizing the clinical diagnostic approach to LRTIs (Diao et al., 2022; Lv et al., 2023). While capable of simultaneously conducting DNA and RNA tests, mNGS remains cost-prohibitive for clinical deployment (Gu et al., 2019). Its diagnostic sensitivity is constrained by both low pathogen reads and overwhelming host DNA background noise, rendering it suboptimal for longitudinal disease surveillance applications (Han, 2022). Unlike mNGS, targeted next-generation sequencing (tNGS) necessitates panel construction through the design of specific primers or probes for preselected pathogens, offering a cost-effective, highly specific, and efficient approach with minimal sample input and reduced human DNA interference (Gaston et al., 2022). And the tNGS method covers the overwhelming majority of respiratory pathogens, ranging from dozens to hundreds (Shi et al., 2023; Zhang et al., 2024). Thus far, the systematic evaluation of tNGS application for pediatric LRTIs has not been sufficiently investigated. This study evaluated the diagnostic utility of tNGS technology in children with LRTIs by assessing its pathogen detection rate and comparing its performance to culture and qPCR methods.

## Methods

### Study setting and patients

**Figure 1:**
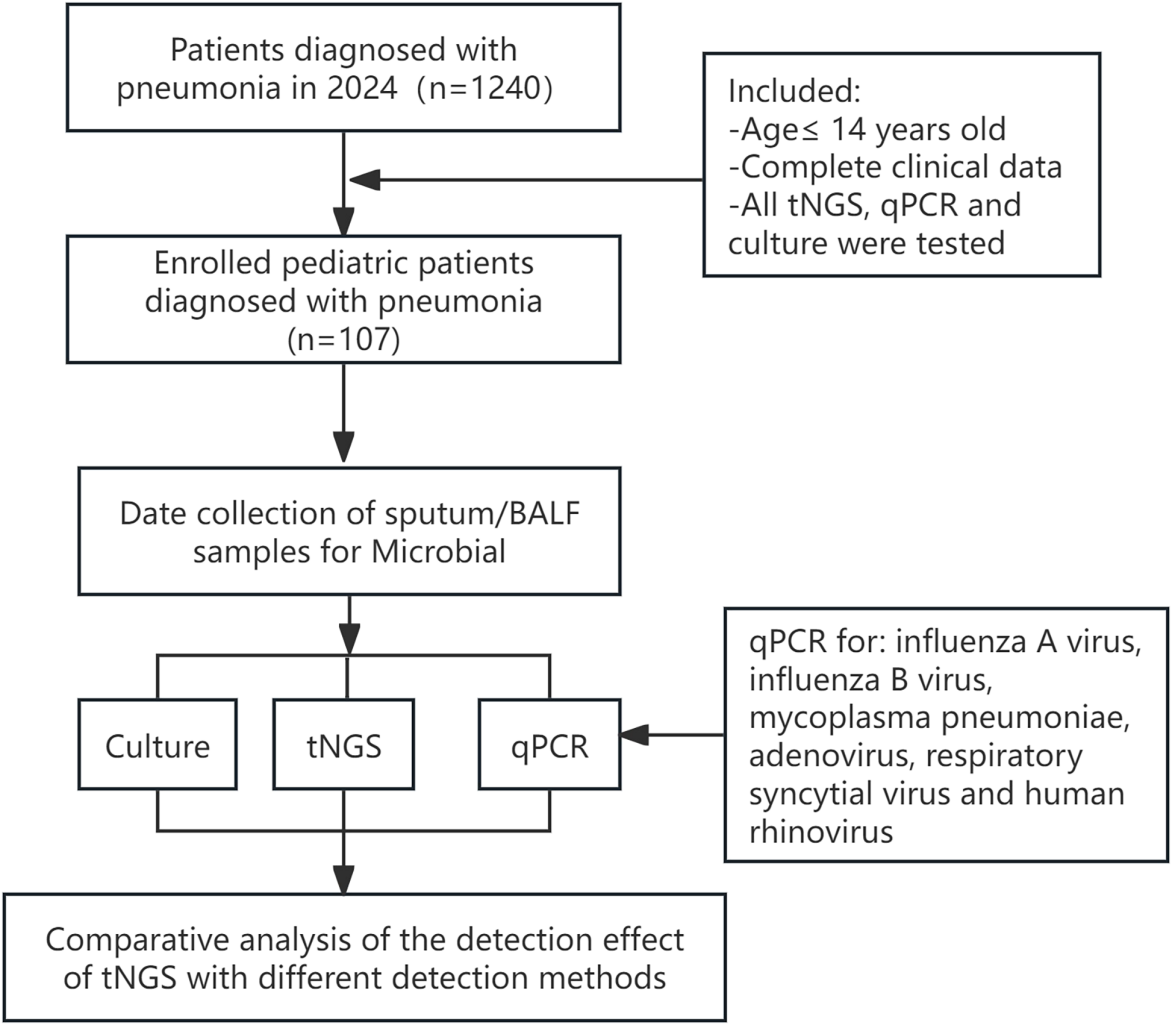
Flowchart of the experimental design for tNGS.

We retrospectively reviewed the medical records of 1240 children hospitalized with confirmed LRTIs at the Maternal and Child Health Hospital of Guangxi Zhuang Autonomous Region, China, from January to December 2024. The study protocol was approved by the Medical Ethics Committee of the Maternal and Child Health Hospital in the Guangxi Zhuang Autonomous Region, and the parents of the pediatric patients signed the informed consent forms. A total of 107 pediatric patients were identified, and their clinical data were collected for analysis (**Figure 1**). The inclusion criteria were as follows: (i) Bronchoalveolar lavage fluid (BALF) or sputum samples obtained from these patients underwent simultaneous testing via tNGS, qPCR, and culture within 72 hours of collection; (ii) from patients aged 14 or younger; (iii) Complete clinical data. Conversely, outpatients and cases with incomplete medical records were systematically excluded from the analysis.

### Collection data and concordance evaluation

In our study, patients who met the inclusion criteria were evaluated by two experienced doctors to determine the relevance of pulmonary infections and potential clinical pathogens. Patient data were collected from the hospital’s electronic health records system and encompassed essential demographic information (gender and age), clinical symptoms, and laboratory examination.

The concordance of the tNGS, qPCR, and culture results with the clinical diagnoses was thoroughly assessed by two experienced doctors. The diagnosis considered the patients’ clinical characteristics, microorganisms tests, tNGS, qPCR, laboratory examination data, imaging findings, and other relevant factors.

### Specimen collection

Fibreoptic bronchoscopy was performed to obtain bronchoalveolar lavage fluid (BALF) for further analysis. Expectorated sputum samples were acquired from children who could expectorate, whereas for those who could not, an attendant nurse collected the samples via nasotracheal suction. BALF and sputum specimens were processed for smear microscopy, staining, and culture. Sample quality was assessed based on cellular composition. A sample was considered to be of good quality when each low-power field (10× magnification) contained at least 25 polymorphonuclear cells and fewer than 10 squamous epithelial cells. In this study, both BALF and sputum samples met these quality criteria. Sputum and BALF specimens were collected in pairs simultaneously for tNGS and qPCR testing.

### Conventional microbiological testing methods

qPCR was performed for *Influenza A virus*, *Influenza B virus*, *Respiratory syncytial virus*, *Adenovirus*, *Human rhinovirus*, and *Mycoplasma pneumonia* using a detection kit (Sansure Biotech Inc., Changsha, China). The Gram staining procedure was conducted using the BaSO Gram staining kit (BaSO Medical Devices Co., Ltd., Zhuhai, China). Specimens were sent to the laboratory for pathogen screening and were cultured on blood agar plates at 37°C in an incubator for 18–24 hours. The isolates were identified using the Zhuhai DL-96II system (Zhuhai DL Biotech Co., Ltd., China) or the VITEK2 Compact system (BioMérieux, Marcy l’Etoile, France). At the same time, additional BALF and sputum specimens were collected and transported to Guangxi Jinyu Medical Examination Center (Nanning, China) for tNGS.

### tNGS assay

Nucleic acids were extracted and purified from the samples using the MagPure Pathogen DNA/RNA Kit (R6672-01B, Magen, Guangzhou, China) following the manufacturer’s instructions. Subsequently, the extracted nucleic acids were analyzed using a multiplex PCR library system (Respiration100TM, KingCreate, Guangzhou, China) to detect 198 respiratory pathogens, including 80 bacteria, 79 viruses, 32 fungi, and seven other pathogens. Upon completion of library preparation, PCR-amplified products were purified through paramagnetic bead-based cleanup to remove residual enzymatic components and primer artifacts. Subsequent amplification was performed using indexed primers with platform-specific adapters and unique barcode sequences. The library and nucleic acid concentration were quantified using Qubit 4.0 Fluorometer (Thermo Scientific) and the Qsep100 Biofragment Analyzer (Guangding Bio). Finally, sequencing through the Illumina MiniSeq platform was then completed by Guangxi Jinyu Medical Examination Center (Nanning, China).

Bioinformatic analyses of tNGS data were performed using the following process. The default settings of fastp v0.20.1 were used to remove sequencing adapters, low-quality reads, reads with excessive N bases, and reads shorter than 35 bp (Chen et al., 2018). The remaining reads were aligned to the human reference (hg38) using Bowtie2 v2.4.1 (Langmead et al., 2009). Subsequently, the reads were aligned against the reference genome database, which included all targeted pathogens and related species within their respective genera. The species genomes were collected and downloaded from National Center for Biotechnology Information’s GenBank, RefSeq, and Nucleotide databases (https://www.ncbi.nlm.nih.gov). The genome assembly level (complete or scaffold) was selected to minimize contamination introduced during assembly, such as host sequences. The detection and identification of specific pathogens were performed in accordance with the Chinese Expert Consensus on the Standardized Application of tNGS in the Diagnosis and Treatment of Infectious Diseases, with additional validation based on our laboratory’s established methodological protocols. Pathogen detection was determined using normalized read counts, expressed as reads per 100,000 (RPhK) (Fu et al., 2025b; Tan et al., 2025). A stringent threshold of RPhK ≥10 was applied to define pathogen positivity, whereas samples with values below this cutoff were considered negative.

### Statistical analysis

Data analysis was performed using IBM SPSS version 25.0. The distribution of categorical variables was described in percentages. For continuous variables following a normal distribution, means and standard deviations are presented in the format: mean ± SD. Continuous variables were analyzed using Student’s t-test or Mann-Whitney U-test, while categorical variables were compared by the χ² test (or Fisher’s exact test when applicable). A P-value < 0.050 was considered statistically significant.

## Results

### Basic clinical characteristics

Based on the inclusion criteria, this study included 107 pediatric patients with LRTIs, of whom 56 were male (52.34%) and 51 were female (47.66%). The median age was three years six months, with the majority (61.68%) being preschoolers. Bronchoalveolar lavage fluid was the primary specimen type analyzed. Most patients presented with non-severe pneumonia, with a mean hospital stay of 8.47 days (SD: 6.45, **Table 1**).

**Table 1:** Clinical characteristics of 107 pediatric patients with pneumonia.

Clinical characteristic	Total (n = 107)	*P-value*
Age		
Median age, $\bar{x}$ (range)	3y6m (23d to14y)	
Neonate (≤ 28 days), n (%)	1 (0.93)	**<0.001**
Infant (29 days- 1 year), n (%)	22 (20.56)	
Preschoolers (1 year-6 years, n (%)	66 (61.68)	
6–14 years old, n (%)	18 (16.82)	
Gender		
Male, n (%)	56 (52.34)	0.494
Female, n (%)	51 (47.66)	
Types of specimens		
Sputum, n (%)	13 (12.15)	**<0.001**
Bronchoalveolar lavage fluid, n (%)	94 (87.85)	
Types of Pneumonia		
Severe pneumonia, n (%)	31 (28.97)	**<0.001**
Non-severe pneumonia, n (%)	76 (71.03)	
ICU admission, n (%)	12 (11.21)	
Length of hospital stay, $\bar{x}$ ± s	8.47 ± 6.45	
Clinical outcomes		
Cure, n (%)	104 (97.20)	**<0.001**
Adverse outcome, n (%)	3 (2.80)	

Bold values in the P-value column indicated statistical significance.

### Assessment of the concordance among tNGS, qPCR, and culture with clinical diagnoses

**Figure 2:**
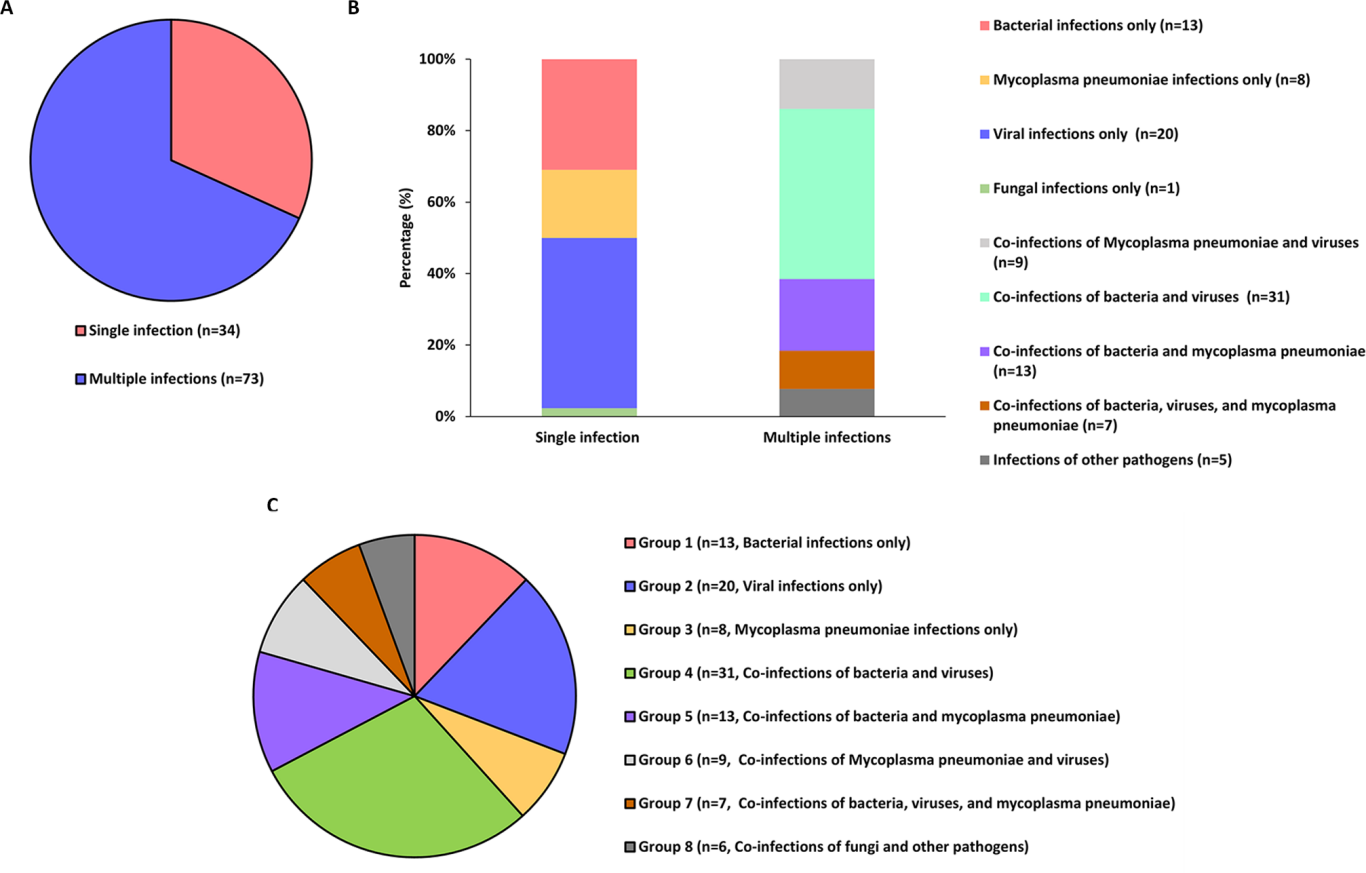
The infection status of 107 patients as well as the evaluation of the concordance between three methods (tNGS, culture, and qPCR) and the clinical diagnostic results. **(A, B)** Categorization of pathogenic infections in patients. **(C)** The subgroup profiles in which the concordance evaluation was conducted.

We assessed the concordance between the three methods and clinical diagnoses across various infection subcategories. Among the 107 enrolled pediatric patients, single infections were identified in 34 cases (31.78%) and multiple infections in 73 cases (68.2%, **Figure 2A**). In single-pathogen infections, Group 2 viral infections were the most prevalent. In comparison, multiple infections were predominantly bacterial-viral co-infections, particularly in Group 4, accounting for the largest subgroup (**Figures 2B, C**). In the analysis of eight different groups, tNGS demonstrated the highest clinical concordance rate (100%) and the lowest rate (66.67%). In comparison, qPCR exhibited concordance rates of 75% (Groups 2 and 3) and 50% (Group 6), while culture showed only 7.69% concordance in group 1. Furthermore, the concordance rate between tNGS results and clinical diagnoses was substantially greater (*P*<0.001) than that between qPCR and culture in Groups 1, 4, 5, 7 and 8 (**Table 2**).

**Table 2:** Differences in clinical concordance between tNGS and the other two methods.

Subgroup	tNGS	qPCR	Culture	*P-value_1_* (tNGS vs. qPCR)	*P-value_2_* (tNGS vs. Culture)
Group 1	76.9%	0	7.7%	**<0.001**	**<0.001**
Group 2	95.0%	75.0%	0	0.182	**<0.001**
Group 3	100.0%	75.0%	0	0.467	**<0.001**
Group 4	100.0%	0	0	**<0.001**	**<0.001**
Group 5	76.9%	0	0	**<0.001**	**<0.001**
Group 6	66.7%	50.0%	0	0.347	**0.009**
Group 7	85.7%	0	0	**0.005**	**0.005**
Group 8	100.0%	0	0	**0.002**	**0.002**

Bold values in the P-value column indicated statistical significance.

### Comparison between tNGS and qPCR for detecting common clinical microorganisms

**Figure 3:**
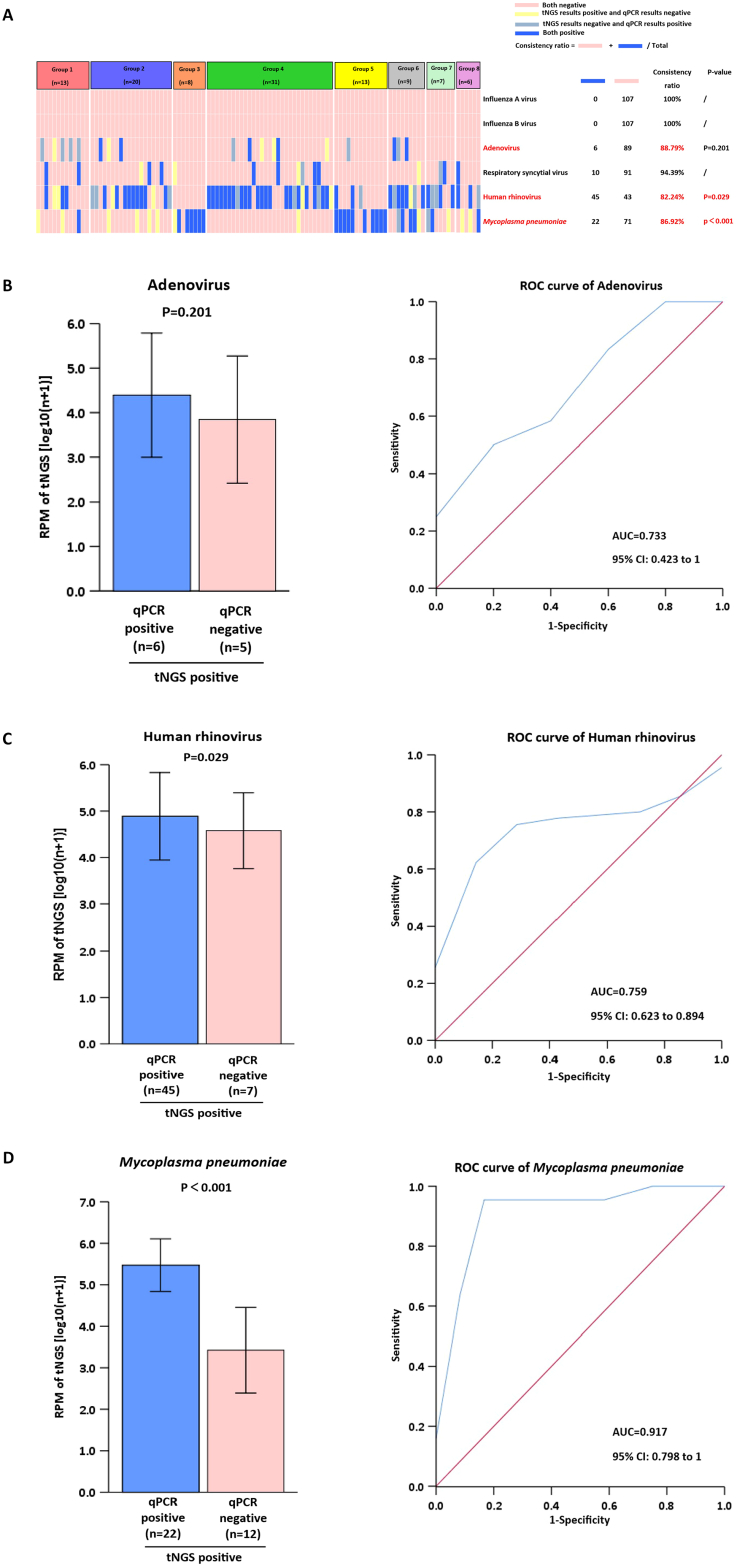
Comparison of tNGS with qPCR for clinical microorganism detection. **(A)** Analysis of Pathogen Detection by tNGS vs. qPCR. **(B-D)** Positive tNGS detections were stratified by qPCR results (positive/negative) for RPM value comparison. ROC analysis used dual-positive (tNGS+qPCR) cases as gold standard.

We selected six clinically relevant microorganisms frequently encountered in diagnostic settings*: Influenza A virus, Influenza B virus, Adenovirus, Respiratory syncytial virus, Human rhinovirus,* and *Mycoplasma pneumoniae*. The detection performance of these pathogens was systematically compared between tNGS and qPCR methodologies. The analysis showed that all results from tNGS and qPCR detection had a concordance rate of up to 80% (**Figure 3A**). *Adenovirus, human rhinovirus*, and *Mycoplasma pneumoniae* were identified with an accuracy rate of ≥ 82.24%. In contrast, *Influenza A virus, Influenza B virus*, and *Respiratory syncytial virus* were detected with a high concordance rate of ≥ 94.39%. Furthermore, we investigated the reasons for the detection rates being below 90%. For *Adenovirus*, the RPM of tNGS in (tNGS^+^qPCR^+^) samples was not significantly different (P>0.05) from that of (tNGS^+^qPCR^−^) samples (**Figure 3B**). For *Human rhinovirus* and *Mycoplasma pneumoniae*, the RPM values for tNGS were significantly higher (P<0.05) in (tNGS^+^qPCR^+^) samples than in (tNGS+qPCR−) samples (**Figures 3C, D**). In comparison to *Human rhinovirus* (AUC=0.759), *Mycoplasma pneumoniae* demonstrated superior differentiation (AUC=0.917).

### Comparison between tNGS and culture for detecting common clinical microorganisms

**Figure 4:**
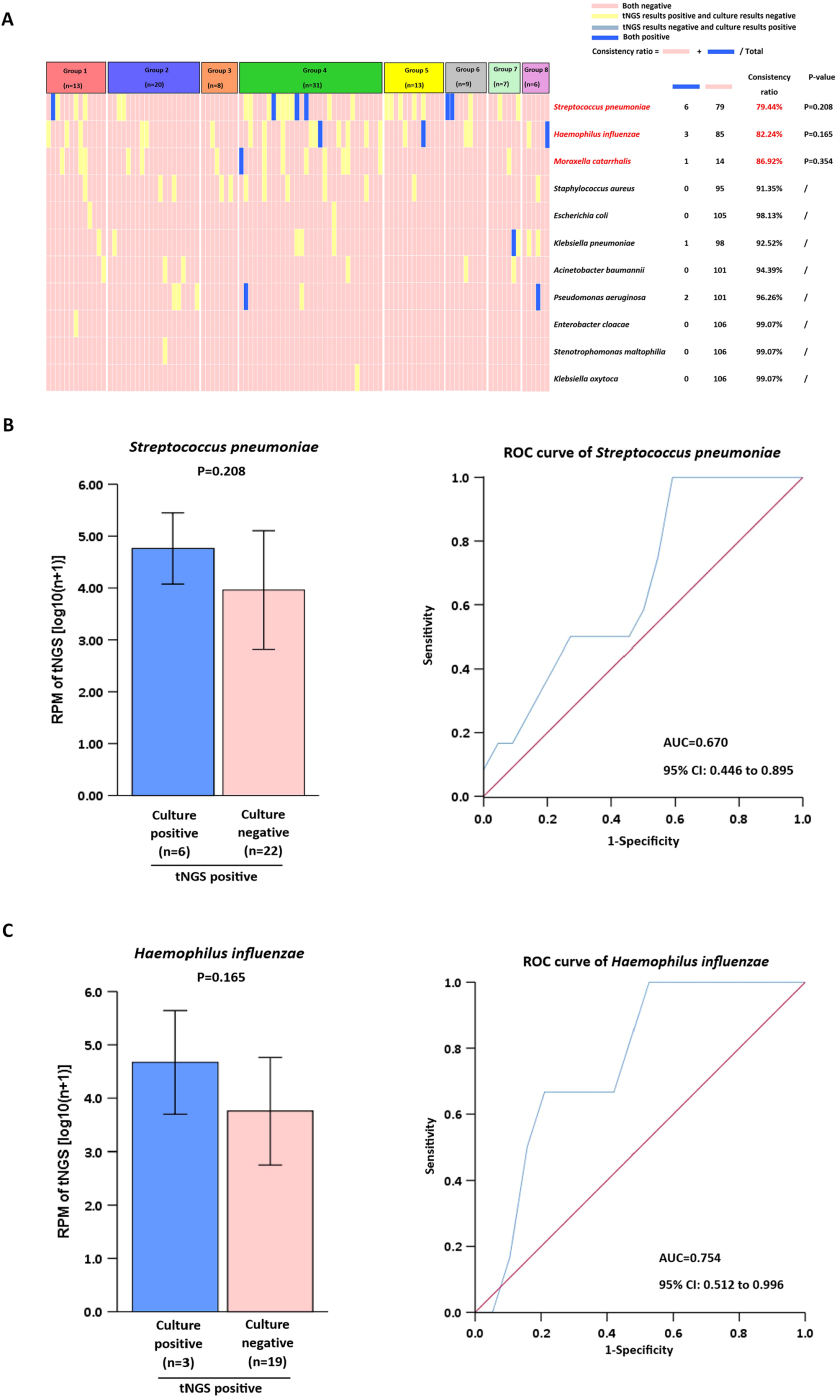
Comparison of tNGS with culture for clinical microorganism detection. **(A)** Analysis of Pathogen Detection by tNGS vs. culture. **(B, C)** Positive tNGS detections were stratified by culture results (positive/negative) for RPM value comparison. ROC analysis used dual-positive (tNGS+qPCR) cases as gold standard.

We selected 11 bacteria frequently found in clinical diagnoses and performed a comparative analysis using tNGS and culture methods. An analysis showed that nearly 70% of tNGS and culture detections had a concordance rate of up to 90% (**Figure 4A**). Common pathogenic microorganisms, including S*treptococcus pneumoniae, Haemophilus influenzae* and *Moraxella catarrhalis*, were identified with an accuracy rate of≥79.44%. In contrast, *Staphylococcus aureus, Escherichia coli, Klebsiella pneumoniae, Acinetobacter baumanni, Pseudomonas aeruginosa, Enterobacter cloacae, Stenotrophomonas maltophilia* and *Klebsiella oxytoca*, were detected with a high concordance rate of ≥91.35%. Additional statistical tests were conducted to analyze the factors contributing to detection rates below 90%. For *Streptococcus pneumoniae* and *Haemophilus influenzae*, the RPM of tNGS in (tNGS^+^culture^+^) samples was not significantly different (P>0.05) from that of (tNGS^+^culture^−^) samples (**Figures 4B, C**). For *Moraxella catarrhalis*, comparisons were not made because of the limited number of samples.

### Clinical characteristics of single infection and multiple infections

Compared to children with multiple infections, those with single infections showed a higher incidence of gastrointestinal symptoms, elevated WBC, and higher LDH levels, all of which were statistically significant (P < 0.05, **Table 3**).

**Table 3:** Clinical charateristicts of single infection and multiple infections.

Factors	Single infection (n=34)	Multiple infections (n=73)	*P-value*
Age,year, $\bar{x}$ (s)	2.9 (2.8)	3.7 (3)	0.219
Neonate (≤ 28 days), n (%)	1 (2.9%)	0 (0.0%)	0.318
Infant (29 days-1 year], n (%)	9 (26.5%)	13 (117.8%)	0.302
Preschoolers (1 year-6 years], n (%)	20 (58.8%)	46 (63.0%)	0.678
6–14 years old, n (%)	4 (11.8%)	14 (19.2%)	0.370
Gener			
Male, n (%)	15 (44.1%)	40 (54.8%)	0.304
Clinical features, n (%)			
Cough	31 (91.2%)	72 (98.6%)	0.058
Expectoration	29 (85.3%)	56 (76.7%)	0.306
Gastrointestinal infection symptoms	10 (29.4%)	7 (9.6%)	**0.009**
Length of hospitalization, day, $\bar{x}$ (s)	8.3 (6.0)	8.5 (6.7)	0.876
Laboratory detection, $\bar{x}$ (s)			
WBC(x10^9^/L)	11.4 (6.5)	9.1 (3.3)	**0.023**
Neu (%)	48.0 (17.8)	47.0 (18.6)	0.805
Lym (%)	42.0 (16.7)	42.0 (16.8)	0.966
Eos (%)	1.9 (1.8)	2.6 (2.6)	0.205
RBC(x10^12^/L)	408.0 (113.7)	386.2 (107.3)	0.349
PLT(x10^9^/L)	4.5 (0.7)	4.5 (0.6)	0.612
Cys-C(mg/L)	0.9 (0.2)	0.9 (0.2)	0.890
CRP (mg/L)	13.8 (11.0)	7.3 (5.1)	0.781
PCT(ng/mL)	0.38 (0.12)	0.23 (0.07)	0.406
AST(U/L)	36.1 (17.5)	30.9 (7.7)	0.092
ALT(U/L)	24.3 (16.3)	20.3 (18.3)	0.393
LDH(U/L)	323.2 (86.3)	272.1 (60.1)	**0.008**
URE (umol/L)	3.4 (1.0)	3.5 (1.2)	0.742
CK(U/L)	93.6 (49.5)	72.0 (38.5)	0.064
CK-MB(U/L)	30.0 (10.7)	26.2 (15.1)	0.329

Bold values in the P-value column indicated statistical significance.

## Discussion

Lower respiratory tract infection (LRTI) is a leading cause of death worldwide, particularly among pediatric populations in low-income regions (Jackson et al., 2023). Microbial infections commonly associated with LRTIs include bacterial, fungal, and viral pathogens, atypical pathogens, and multiple concurrent infections (Liu et al., 2018). This study shows that bacteria, fungi, and viruses are responsible for LRTIs in children, and 68.3% of these infections involve multiple pathogens, aligning with prior research. Therefore, early and accurate pathogen identification can reduce morbidity and mortality (Zhang et al., 2022).

This study demonstrates that viral infections are the most prevalent in single pathogen infections, whereas bacterial-viral co-infections predominate in polymicrobial infections. Of particular clinical significance were the recognition that viral infections may predispose to secondary bacterial pneumonia through mechanisms of epithelial damage and immune modulation (Hanada et al., 2018; Manohar et al., 2020). These highlight that rapid viral identification and early initiation of antiviral therapy significantly influence disease progression in respiratory viral infections. The diagnosis of respiratory viruses has evolved significantly since the introduction of multiplex PCR panels and other molecular techniques, which allow rapid simultaneous detection of multiple respiratory viruses (Clark et al., 2023). For example, mNGS, tNGS, and hybridization have a short TAT, high sensitivity and specificity, and high accuracy compared with conventional phenotypic methods (Wang et al., 2019).

The application of tNGS technologies for pathogen identification is a burgeoning field, with optimal practices still under active investigation. Recently, tNGS has been applied to detect respiratory tract pathogens (Ma et al., 2024; Qin et al., 2025). This study found that tNGS results showed a concordance rate of >65% with clinical diagnoses in pediatric patients with LRTIs. Consistent with previous studies, tNGS showed a concordance rate of more than 70% with clinical diagnoses in pneumonia patients (Zhang et al., 2024). Additionally, the study demonstrates that tNGS shows the highest diagnostic concordance among available methods in detecting Mycoplasma pneumoniae, viral, and fungal pathogens. Notably, since Mycoplasma pneumoniae and viruses are the most prevalent causes of pediatric LRTIs (Liu et al., 2018); tNGS proves particularly valuable for diagnosing such infections in children.

While our study demonstrates superior concordance between tNGS and clinical diagnoses, discordant cases warrant careful consideration, as they may reflect either technical limitations or clinically meaningful discrepancies. tNGS may detect low-abundance nucleic acids that do not represent true infection from colonizers such as Streptococcus pneumoniae in the nasopharynx (Chen et al., 2024). This scenario is particularly relevant in pediatric populations where asymptomatic carriage rates are high. Furthermore, even after clinical recovery, non-infectious pathogen fragments or residual genetic material may persist, potentially causing tNGS positivity in the absence of active infection. This aligns with studies on Adenovirus detection, where tNGS may overcall positives in some cases (Huang et al., 2024). Therefore, to mitigate misinterpretation, we highlight the importance of integrating tNGS results into the clinical context—for example, through correlation with white blood cell counts, procalcitonin levels, imaging data, and clinical manifestations.

The targeted enrichment process enhances the sensitivity of tNGS, enabling the detection of pathogens that are undetectable by qPCR methods (Zhao et al., 2021). Examples include *Adenovirus, human rhinovirus, Mycoplasma pneumoniae, Streptococcus pneumoniae*, and *Haemophilus influenzae*. The superior detection capability of tNGS over qPCR methods for pathogenic bacteria may originate from their distinct limits of detection and varying genome coverage rates for different pathogens (Chen et al., 2024). Our data indicate a high concordance rate (≥94.39%) for the detection of *Influenza A, Influenza B*, and *RSV*, which is consistent with previous researches (Zhang et al., 2024; Wang et al., 2025). The near-perfect concordance in detecting I*nfluenza A, Influenza B*, and *RSV* likely stems from the high sensitivity of tNGS to elevated viral loads during acute infections, combined with the robustness of amplification-based methods for these RNA viruses. However, the lower concordance for Adenovirus demands careful interpretation. The lack of significant RPM difference between tNGS+/qPCR+ and tNGS+/qPCR- samples suggests tNGS may detect low-level *Adenovirus* DNA below the clinical sensitivity threshold of qPCR or from non-replicative virus fragments (Chen et al., 2024). This highlights a critical challenge: distinguishing clinically relevant infection from incidental carriage or residual nucleic acid, particularly for persistent viruses like *Adenovirus*.

*Mycoplasma pneumoniae* and *human rhinoviruses* are common pathogens causing infections in hospitalized children (Yun KW et al., 2022). tNGS demonstrated substantial clinical utility in identifying *Mycoplasma pneumoniae* and the *human rhinoviruses* frequently detected in respiratory tract infections (Fu et al., 2025a). Several studies have shown that using qPCR or tNGS to monitor pathogen dynamics continuously may help clinicians better assess patient status (Gibert et al., 2024; Zhang et al., 2024). In our study, for *HRV* and *M. pneumoniae*, the significantly higher RPM values in concordantly positive samples (tNGS+/qPCR+) than in tNGS+/qPCR− samples indicate that RPM magnitude correlates with qPCR positivity. This suggests that RPM could serve as a quantitative measure, particularly for M. pneumoniae (AUC=0.917), effectively distinguishing true positives. The lower AUC for HRV (0.759) implies greater overlap in RPM ranges between true positives and potential false positives/background, possibly reflecting that the high genetic diversity of HRV strains affects primer/probe binding efficiency in qPCR or leads to variable replication kinetics. Thus, while tNGS reliably detects viral presence, RPM thresholds may serve as necessary adjuncts for clinical interpretation.

For lower respiratory tract infections, the traditional culture method has several shortcomings, such as a low positive rate, a long turnaround time, and a limited range of detectable pathogens (Foschi et al., 2021). Studies have shown that tNGS outperforms culture methods in bacterial detection (Chen et al., 2025; Li et al., 2025). The results of this study demonstrate that although the detection concordance rate for common bacteria (e.g., *Streptococcus pneumoniae* and *Haemophilus influenzae*) exceeded 78%, tNGS exhibited a significantly higher positive detection rate compared to conventional culture methods, consistent with previous research. The superior sensitivity of tNGS in detecting more infections compared to culture methods may result from variations in detection thresholds across different pathogens. However, when designing tNGS probes that prioritize genus-level sequences and inadequately cover species-specific sequences, this approach demands higher caution. It often leads to failed species-level categorization and a higher detection limit (Gaston et al., 2022). Furthermore, although tNGS exhibits a high detection rate, it lacks the ability to perform antimicrobial susceptibility testing (AST). This gap may delay optimal antibiotic initiation, particularly with fastidious organisms or failed empirical treatments. In such cases, clinicians must rely on supplemental culture results or molecular detection of resistance genes, both of which may require additional time and resources. Future advances, such as machine learning–based prediction of resistance from genomic data or rapid phenotypic AST platforms, may help bridge this gap.

Several studies have identified polymicrobial infections as one of the key characteristics of pediatric LRTIs (Ademhan Tural et al., 2022; Li et al., 2023; Rouze et al., 2025). Several studies highlight tNGS’s potential to supplement routine diagnostic methods in cases of multiple pathogen co-infection (Tan et al., 2025; Xiao et al., 2025). Consistent with previous studies, we identified polymicrobial infections in 76 patients. In our study, tNGS outperformed other methods by achieving significantly higher detection efficiency. Furthermore, we compared the clinical characteristics between patients with single infection and those with multiple infection. The results showed that no indicators were significantly associated with multiple infection, which further demonstrates the important role of tNGS in identifying and diagnosing mixed infections.

This study had several limitations. First, since this study was a single-center retrospective investigation, its findings may be subject to selection bias. Thus, validation through future research with larger samples and prospective designs is necessary. Secondly, the detection results lack an assessment of clinical prognosis. Future prospective studies should establish a direct association between tNGS findings and patient outcomes, such as mortality rates and length of hospital stay. Thirdly, the range of commercially available PCR kits was limited, particularly as common respiratory pathogens had not been validated using PCR methodology. Furthermore, some of the patients in our study received antibiotic therapy, which affected the diagnostic performance of the culture method. And finally, it is often unclear whether microbes identified through tNGS represent contaminants, colonizers, or pathogens. These distinctions require further investigation, particularly in lower respiratory tract infections.

## Conclusion

Although tNGS is more sensitive than both qPCR and culture in detecting pathogens causing pediatric LRTIs, it cannot replace drug sensitivity results. Our data suggest that the tNGS results demonstrated higher concordance with the clinical diagnosis. tNGS is highly applicable for diagnosing pathogens in pediatric patients with severe, mixed, or refractory infections. tNGS technology has demonstrated diagnostic potential to complement routine diagnostics, particularly for opportunistic pathogens and mixed infections, or for cases with negative qPCR and culture testing.

## Data Availability

The original contributions presented in the study are included in the article/supplementary material. Further inquiries can be directed to the corresponding author.
